# Reexamining Sample Size Requirements for Multivariate, Abundance-Based Community Research: When Resources are Limited, the Research Does Not Have to Be

**DOI:** 10.1371/journal.pone.0128379

**Published:** 2015-06-09

**Authors:** Frank L. Forcino, Lindsey R. Leighton, Pamela Twerdy, James F. Cahill

**Affiliations:** 1 Western Carolina University, Geosciences and Natural Resources Department, 331 Stillwell Building, Cullowhee, NC, United States of America, 28723, (828) 227–7367, fax: (828) 227–7647; 2 University of Alberta, Earth & Atmospheric Sciences, 1–26 Earth Sciences Building, Edmonton, AB, Canada, T6G 2E3; 3 University of Alberta, Department of Biological Sciences, CW 405, Biological Sciences Building, Edmonton, AB, Canada, T6G 2E9; Instituto Español de Oceanografía, SPAIN

## Abstract

Community ecologists commonly perform multivariate techniques (e.g., ordination, cluster analysis) to assess patterns and gradients of taxonomic variation. A critical requirement for a meaningful statistical analysis is accurate information on the taxa found within an ecological sample. However, oversampling (too many individuals counted per sample) also comes at a cost, particularly for ecological systems in which identification and quantification is substantially more resource consuming than the field expedition itself. In such systems, an increasingly larger sample size will eventually result in diminishing returns in improving any pattern or gradient revealed by the data, but will also lead to continually increasing costs. Here, we examine 396 datasets: 44 previously published and 352 created datasets. Using meta-analytic and simulation-based approaches, the research within the present paper seeks (1) to determine minimal sample sizes required to produce robust multivariate statistical results when conducting abundance-based, community ecology research. Furthermore, we seek (2) to determine the dataset parameters (i.e., evenness, number of taxa, number of samples) that require larger sample sizes, regardless of resource availability. We found that in the 44 previously published and the 220 created datasets with randomly chosen abundances, a conservative estimate of a sample size of 58 produced the same multivariate results as all larger sample sizes. However, this minimal number varies as a function of evenness, where increased evenness resulted in increased minimal sample sizes. Sample sizes as small as 58 individuals are sufficient for a broad range of multivariate abundance-based research. In cases when resource availability is the limiting factor for conducting a project (e.g., small university, time to conduct the research project), statistically viable results can still be obtained with less of an investment.

## Introduction

Community ecologists commonly perform multivariate techniques (e.g., ordination, cluster analysis) to assess patterns and gradients of taxonomic variation [1–6]. Due to the enormous number of individuals in most ecological communities, ecologists typically rely on a collected sample that is representative of the complete natural system as opposed to collecting everything within a natural system [7–15]. This fundamental unit of sampling must contain a sufficient number of individuals; otherwise it may misrepresent the natural system leading to erroneous conclusions. For the purposes of this paper, the fundamental unit of sampling or the number of individuals per sample will be called sample size—the total number of individual specimens comprising one row of data in a taxon-sample matrix used for multivariate community analysis.

Determining a minimum representative sample size at which the results of a community analysis would be unchanged from those obtained with larger sample sizes has thus been a major practical concern for ecologists [16–20]. Here, we determine the smallest required sample size at which a statistically robust result can be achieved using multivariate statistical techniques.

Although researchers must collect a sample size that is large enough to be representative, once that sample size has been obtained, additional samples should not alter the outcome of a multivariate analysis, and such additional material can be considered a form of over-sampling. When the cost—in time, money, or other resources—of collecting or identifying individuals within a sample is nominal, oversampling may not be an issue. However, there are many situations in which oversampling results in significant increased costs, with little improvement in the ability to answer specific ecological questions. Thus, it is important to provide ecologists with guidelines regarding when smaller sample sizes can be used and still retain a statistically robust analysis.

Three instances when it would be beneficial to know that a smaller sample size is as statistically robust as larger sample sizes are (1) if the researcher is limited by funds. (2) Time may be a limiting factor in some research projects for a number of possible reasons. One common cause for a limit on time is when conducting a research project with an undergraduate student [21,22]. In addition, professors in academic settings typically only have four to five months out of the year with no teaching requirements. Furthermore, there may be some types of data that can only be sampled during a short time span. (3) There are cases in which data have already been collected by previous researchers. Those data may previously have been discarded from subsequent meta-analyses because the sample size was believed to be too small. So, if we can demonstrate that smaller sample sizes are sufficient, it could open up the use of these legacy data for additional ecological analyses.

The present study is aimed at the situations in which the above-discussed factors are limited and thus the project may benefit from knowing if smaller representative samples are as statistically robust as larger sample sizes. Here, our goal is to determine the smallest sample size that can be collected and used in multivariate, abundance-based ecological research.

Previous research has determined appropriate sample sizes for many types of ecological research by examining the probability of acquiring species that comprise some proportion of a sample with 95% confidence [23,16,24,17,18,25]. In other words, how likely is a sample to contain taxon Z if a sample size of Y individuals is collected? However, determining sample size in this manner is most often used for the purpose of assessing and comparing diversity among samples. Although this is important, these approaches do not take into account ecological relationships among sampling units or taxa. Other workers have compared multivariate results of plots or samples collected at different sample sizes to determine which sample size perform best using multivariate techniques [26,12,27,19], but no one has examined this on a large scale, using multiple types of ecological data and multiple published community datasets. Moreover, determining which sample size provides maximum information may not actually be relevant to the goal of a community ecology study. For many studies, the question of interest is how different communities relate to each other and how or whether a given environmental variable influences the communities. For such studies, an important question is how small can the sample size can be and still produce robust multivariate statistical results?

Forcino (2012) conducted a meta-analysis of 30 fossil community datasets and found that a median sample size of 50 individuals is sufficient for producing robust multivariate statistical results when conducting abundance-based research. Here, using methods similar to Forcino (2012), we examine 396 datasets, 44 previously published, modern datasets and 352 created datasets. Using meta-analytic and simulation approaches, the present research seeks (1) to determine minimal sample sizes required to produce robust multivariate statistical results when conducting abundance-based community ecology research. Furthermore, we seek (2) to determine the dataset parameters (i.e., evenness, number of taxa, number of samples) that require larger sample sizes, regardless of resource availability. We recognize that these specific forms of multivariate analyses are not the only ones used; however, they are used commonly in community ecology, and thus using this novel approach can provide important insight for ecologists [7,8,4]. Evidence relevant to goal (1) will provide ecologists with a more accurate estimate of the minimum representative number of individuals for multivariate research, and lead to a better use of resources. Accomplishing goal (2) will inform ecologists when greater resources are necessary to obtain a statistically robust sample of a community.

## Materials and Methods

### Previously published datasets

In order to test if smaller samples sizes could produce the same results originally obtained from previous research, 44 real datasets were acquired from the ecological literature (Table 1). These datasets comprise a range of numbers of taxa (3 to 421) and samples (4 to 445), were from different taxonomic groups, different geographic locations, different environments, and published by different authors (Table 1). In addition, 18 datasets were gathered from one meta-analysis [28]. Twenty additional studies from other journals were also used in the analysis (Table 1). Working backward through time of publication, we selected and subsampled the first 44 datasets that had median sample sizes of at least 20 individuals.

**Table 1:** A list of the 44 previously published datasets (some of the citations contain multiple datasets) including original characteristics of the complete dataset.

Citation	Median Sample Size	Number of Samples	Number of Taxa	Mean Evenness	Environment	Primary Taxonomic Group	Geographic Location
Beehler 1983 [44]	97	8	31	0.72	Forest	Birds and plants	Papua New Guinea
Arthur et al. 1976 [45]	85	38	17	0.74	Lake	Parasites	Yukon, Canada
Cause et al. 2011 [46]	20	43	53	0.74	Subtidal marine	Parasites	Dumont d’Urville Sea (East Antarctica)
Wong et al. 2004 [47]	24812	12	13	0.46	Fresh water streams	Invertebrates	Kent, Uk, and Mississippi, USA
VanNimwegen et al. 2008 [48]	75	4	7	0.69	Grasslands	Prairie dogs	Kansas, USA
Ieno and Bastido 1998 [49]	853	7	13	0.75	Benthic marine	Bivalves and ploychaetes	Samborombon Bay, Argentina
Kinnunen and Tiainen 1999 [50]	147	40	7	0.59	Farmland	Beetles	Finland
Nicolaidou et al. 2006 [51]	890	18	48	0.64	Benthic lagoon	Bivalves	Ionian Sea, Greece
Arai and Mudry 1983 [52]	114	17	53	0.83	River	Fish and parasites	British Columbia, Canada
Peres 1997 [53]	110	12	12	0.94	Forest	Primates	Brazil
Dahle et al. 1998 [54]	944	15	421	0.70	Benthic brackish	Marine invertebrates	Pechora Sea, Russia
Repecka and Mileriene 1991 [55]	511	19	20	0.95	Marine	Fish	Kursia Bay, Lithuania
Hughes and Thomas 1971 [56]	94	16	16	0.69	Benthic Estuary	Bivalves	Prince Edward Island, Canada
Hughes and Thomas 1971 [56]	76	21	18	0.67	Benthic Estuary	Bivalves	Prince Edward Island, Canada
Hughes and Thomas 1971[56]	235.5	14	14	0.51	Benthic Estuary	Bivalves	Prince Edward Island, Canada
Hughes and Thomas 1971[56]	648	51	51	0.72	Benthic Estuary	Bivalves	Prince Edward Island, Canada
Ryu et al. 2011 [35]	4939	7	36	0.53	Benthic marine to brackish	Benthic animals	Incheon North Harbor, Korea
Skrodowski and Porowski 2000 [57]	210	25	22	0.73	Pine forest	Beetles	Poland
Snow and Snow 1971 [58]	146	13	65	0.76	Neotropical forest	Birds	Trinadad
Snow and Snow 1988 [59]	234	7	12	0.50	Mixed terrestrial	Birds and plants	England
Snow and Snow 1971[58]	1674	9	35	0.70	Neotropical forest	Birds	Trinadad
Ulrich and Zalewski 2006 [60]	145	11	17	0.76	Lake Islands	Beetles	Multiple
Dechitar 1972 [61]	338	31	144	0.93	Lake	Parasites	Ontario, Canada
Anderson et al. 2011 [62]	850.5	42	39	0.52	Northern mixed prairie	Grassland plants	Montana, USA
Anderson et al. 2011[62]	9.5	10	6	0.80	Northern mixed prairie	Grassland plants	Montana, USA
Anderson et al. 2011[62]	261	25	15	0.50	Northern mixed prairie	Grassland plants	Montana, USA
Anderson et al. 2011[62]	52	29	14	0.50	Northern mixed prairie	Grassland plants	Montana, USA
Anderson et al. 2011[62]	29	42	31	0.55	Northern mixed prairie	Grassland plants	Montana, USA
Anderson et al. 2011[62]	35	39	17	0.44	Northern mixed prairie	Grassland plants	Montana, USA
Anderson et al. 2011[62]	29	42	41	0.63	Northern mixed prairie	Grassland plants	Montana, USA
Anderson et al. 2011[62]	118	37	46	0.50	Northern mixed prairie	Grassland plants	Montana, USA
Anderson et al. 2011[62]	30	37	43	0.67	Northern mixed prairie	Grassland plants	Montana, USA
Anderson et al. 2011[62]	248	41	46	0.60	Northern mixed prairie	Grassland plants	Montana, USA
Anderson et al. 2011[62]	573	42	37	0.47	Northern mixed prairie	Grassland plants	Montana, USA
Anderson et al. 2011[62]	53	41	27	0.71	Northern mixed prairie	Grassland plants	Montana, USA
Anderson et al. 2011[62]	20	41	30	0.72	Northern mixed prairie	Grassland plants	Montana, USA
Miller et al. 2011 [63]	6431	68	117	0.66	Marine	Fish	Pacific coast, USA
Petraitis et al. 2009 [64]	301	60	3	0.67	Intertidal	Bivalves and algae	Maine, USA
Ramesh et al. 2010 [65]	132	95	334	0.77	Tropical terrestrial	Plants	Karnataka, India
Stevens et al. 2011 [66]	33	280	155	0.90	Grasslands	Plants and bryophytes	Atlantic coast, Europe
Stevens et al. 2011 [66]	51	40	100	0.93	Grasslands	Plants and bryophytes	Atlantic coast, Europe
Stevens et al. 2011 [66]	52	445	355	0.95	Grasslands	Plants and bryophytes	Atlantic coast, Europe
Ulrich and Gotelli 2010 [28]	248	6	25	0.77	River	Fish	British Columbia, Canada
Ulrich and Gotelli 2010 [28]	495	8	99	0.88	Lake Islands	Beetles	Multiple

The environment refers to the broadest environment from which samples were collected. The primary taxonomic group is broadest category of the most abundant groups in dataset. This list is meant to show the diversity of the types of data included in the analysis.

### Created datasets

#### Gradient Analysis

To determine if smaller sample sizes are sufficient for datasets with properties outside the ranges of the 44 previously published datasets examined, we created 352 datasets. Our goal was not to examine all possible datasets that could exist in nature, but to complement the 44 previously published, real datasets by creating datasets with parameters that did not consistently exist among any the 44 real datasets (e.g., sample sizes larger than 1000 individuals per sample). This allowed us to have a greater number of datasets with certain parameters (e.g., high evenness) to further gauge the required sample size for those datasets or samples with those parameters. Similar to the real datasets, the created datasets contained a range of numbers of taxa and samples (Table 2). The number of datasets created balances the computer time needed to subsample each dataset with the new information gained from adding more datasets.

**Table 2:** A list of the 220 created datasets with simulated abundance structure along with the characteristics of the complete dataset prior to subsampling.

Datasets	Number of samples	Number of taxa	Gradient Size	Median sample size
1–10	15	15	1000	8418
11–20	15	15	5000	41528
21–30	20	20	100	178
31–40	20	20	5000	58014
41–50	20	30	100	278
51–60	20	40	100	256
61–70	20	40	100	356
71–80	20	50	100	255
81–90	20	50	100	493
91–100	25	100	100	936
101–110	30	20	100	194
111–120	30	60	100	376
121–130	40	20	100	169
131–140	50	20	100	181
141–150	50	50	100	474
151–160	50	50	5000	151434
161–170	50	75	100	761
171–180	50	100	100	982
181–190	50	200	100	2037
191–200	75	50	100	484
201–210	100	50	100	468
211–220	200	50	100	462

This list is meant to show how the dataset were structured, and the differences among the datasets. The number of samples, number of taxa, and gradient size were controlled for in the simulation. The median sample size was an output result of the randomized simulation, although it was influenced by the controlled parameters.

Two protocols were used for the constructions of the created datasets: 220 datasets were created using a simple random simulation, and 132 were created by keeping each sample at a constant 200 individuals and systematically altering the abundance structure of each sample. We explain each of these two processes in detail below.

For the first 220 created datasets, we followed a simple random selection protocol to create the datasets in order to obtain additional samples that were comparable to the previously published datasets. Datasets were simulated by first randomly creating a normal distribution of abundances for each taxon across a hypothetical gradient (S1 Fig). Each normal distribution for each taxon was created based on a randomly chosen mean, randomly chosen standard deviation, and randomly chosen maximum possible abundance. The resulting distribution represents the range along an ecological or environmental gradient within which each simulated taxon is located. Each simulated taxon has a peak possible abundance, and areas along the gradient where the taxon is less likely to be found (the tails of the normal distribution with a lower abundance). For example, if the hypothetical gradient represents water depth, the randomly selected mean of the normal distribution for Taxon A represents the optimal depth at which Taxon A lives, and therefore, maximum peak of abundance. The tails of the normal distribution represent the most extreme conditions (shallowest and deepest depths) in which Taxon A lives, with abundances declining from the peak to each tail.

Sample locations for each taxon were randomly selected along the environmental gradient (S1 Fig), simulating random sampling of a gradient in the field under circumstances where continuous or interval sampling is not possible. For example, if the gradient was 100 units long (the total unit length is an arbitrary value representing the complete gradient length), a unique number from 1 to 100 was randomly selected for each sample, which represents the sampling locations along the gradient. This process was repeated for the number of taxa selected for that particular dataset (Table 2). At each of these sampled points, all of the taxonomic distributions that cross that point are included in that sample. Taxon abundances equal the height of the curve of each taxon’s normal distribution at that point along the gradient (S1 Fig). This process was repeated for each of the 220 datasets (Table 2).

#### Effects of evenness, samples, and taxa

Additionally, 132 datasets were created with the intention of deliberately generating more extreme differences among complete and subsampled datasets. In each dataset, we selected the abundances (as opposed to randomly generating the abundances) for each taxon in each sample. The other differences among each dataset were the number of samples (i.e., the number of collected samples in a dataset that would equate to one row in a taxon by sample matrix), numbers of taxa, and evenness. These three variables were selected because they commonly vary from study to study, and two of them are among the most basic ecological measures (i.e., number of taxa and evenness).

This selected, systematic creation of datasets often led to datasets with rank abundance distributions and absolute abundances that are rare in the literature, but which might facilitate identification of the conditions under which larger sample sizes would be necessary to capture the multivariate results of a dataset. We also systematically varied the number of samples, richness, and evenness to examine these three variables as possible influences on the required sample size for community research.

Each sample of each of these 132 datasets contained 200 individuals (For complete list of these datasets see S1 R Data). A sample size of 200 was chosen so that five subsample proportion sizes (100, 50, 20, 10, 5) could be produced that represent a range of smaller sample sizes, for comparison with the complete sample.

These 352 datasets were not meant to be all encompassing in terms of creating all possible datasets a researcher might collect in the field. They were meant to add datasets that complement the 44 previously published datasets by providing additional evidence if smaller sample sizes are sufficient for abundance-based ecological research and if there are any conditions (e.g., high evenness) when larger samples sizes are needed.

### Statistical Analyses

In order to replicate going into the field and collecting smaller sample sizes than used previously in each study, using R 2.14 [29], each sample within each taxon-sample matrix (previously published and created) was randomly subsampled without replacement to five proportional sizes: 50%, 25%, 10%, 5%, and 2.5% of the total number of individuals in the original sample. For each of the subsampled proportions of each taxon-sample matrix, 1000 subsampled matrices were constructed for a total of 5000 subsampled matrices for each dataset. Each of the 5000 subsampled matrices was statistically compared to the original 100% taxon-sample matrix using two multivariate statistical methods.

(1) Using the vegan package in R 2.14 [30], Mantel Tests of correlation were performed between the Bray-Curtis dissimilarity matrices (measures of the differences between each object in a taxon-sample matrix) of subsamples and corresponding complete datasets. We conducted pilot trials with three datasets to determine if various dissimilarity measures affect the resulting comparison goodness-of-fit statistics. Regardless of distance measure (Bray-Curtis, Euclidian, City-block, or Raup-Crick), results were the same.

The Mantel Test tests the similarity of two matrices of dissimilarity indices by permuting each of the elements in the dissimilarity matrix 999 times, to derive a distribution of correlation values [31,5,32]. The resulting R-statistic is analogous to the Pearson’s Product Moment Correlation Coefficient (r); with increasingly similar data matrices, the Mantel R-statistic will approach 1.

(2) For each of the datasets and subsamples, non-metric multidimensional scaling (NMDS) ordinations of the samples were performed using the Bray-Curtis dissimilarity index [33,2,3,34]. All ordinations were run examining the taxonomic distributions among samples with two dimensions with “autotransform = false” in the vegan package in R, specifically using the function “metaMDS()”.

Procrustean Randomization Tests (PROTEST) were performed comparing procrustes transformed ordinations of the subsampled and corresponding complete datasets [35,36]. NMDS does not always assign the maximum explanation of variation in the ordination space to the first axis. Moreover, two different ordinations might not appear to be similar at first because they are close reflections to each other in ordination space. To address these possibilities, the first step in PROTEST is to perform a Procrustes transformation, which minimizes the sum-of-squares deviations between the two ordination results through translation, reflection, rotation, and dilation. Thus, the two ordination results are reoriented such that they are aligned as closely as possible in ordination space, which permits a more accurate assessment of similarity. The residuals between the two ordinations post-transformation are calculated and produce the m^2^-value. The m^2^-value is similar to the r-value resulting from a Pearson’s Product Moment Correlation; the closer m^2^ is to 1, the more similar the two ordinations. Subsequent to the Procrustes transformation, PROTEST randomly permutes the ordination scores for all samples for 999 iterations, and the m^2^-value is calculated for each iteration; a realized p-value, indicating the significance of the m^2^-value, is then calculated by determining the percentage of iterations in which the m^2^-values from the randomized iterations are greater than the m^2^-value for the actual dataset.

## Results

### Previously published datasets

**Fig 1:**
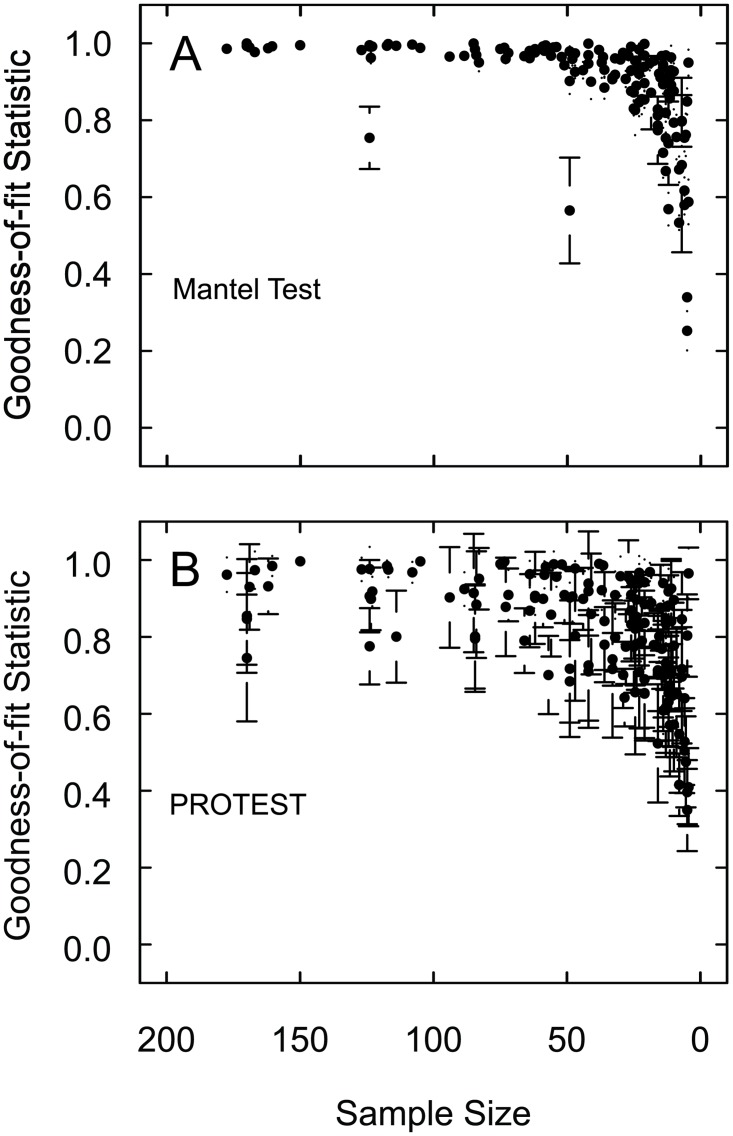
(a) Mantel test and (b) PROTEST comparisons for each of the five median subsample sizes for each of the 44 previously published datasets. Each point (black circles) represents the mean, plus and minus one standard deviation, (a) R-statistic and (b) m^2^-values for the 1000 subsamples of one datasets at one sample size. There are five points for each dataset—one for each of the subsample sizes.

With the exception of one dataset, the Mantel Test R-statistics were greater than R = 0.88 for all sample sizes greater than 28 individuals (Fig 1a). When the median sample size is less than 28 individuals the R-statistics decrease rapidly. The one dataset that is below R = 0.88 was Ryu et al. (2011), which contained primarily ostracods with median sample size of 4939 [37]. The Procrustean Randomization Test (PROTEST) m^2^-values were consistently above m^2^ = 0.76 at median sample sizes greater than 58 (Fig 1b); the m^2^-values decrease rapidly at smaller samples. The threshold values (R = 0.88, m^2^ = 0.76) are based on shifts in values of the goodness-of-fit statistics between a plateau of similar values to a rapid decrease in values. These breaking points are assumed to distinguish those sample sizes sufficient to produce the same results, based on the constant, relatively high goodness-of-fit statistics, from those sample sizes that fail to produce the same results as the complete data sets.

### Created datasets

**Fig 2:**
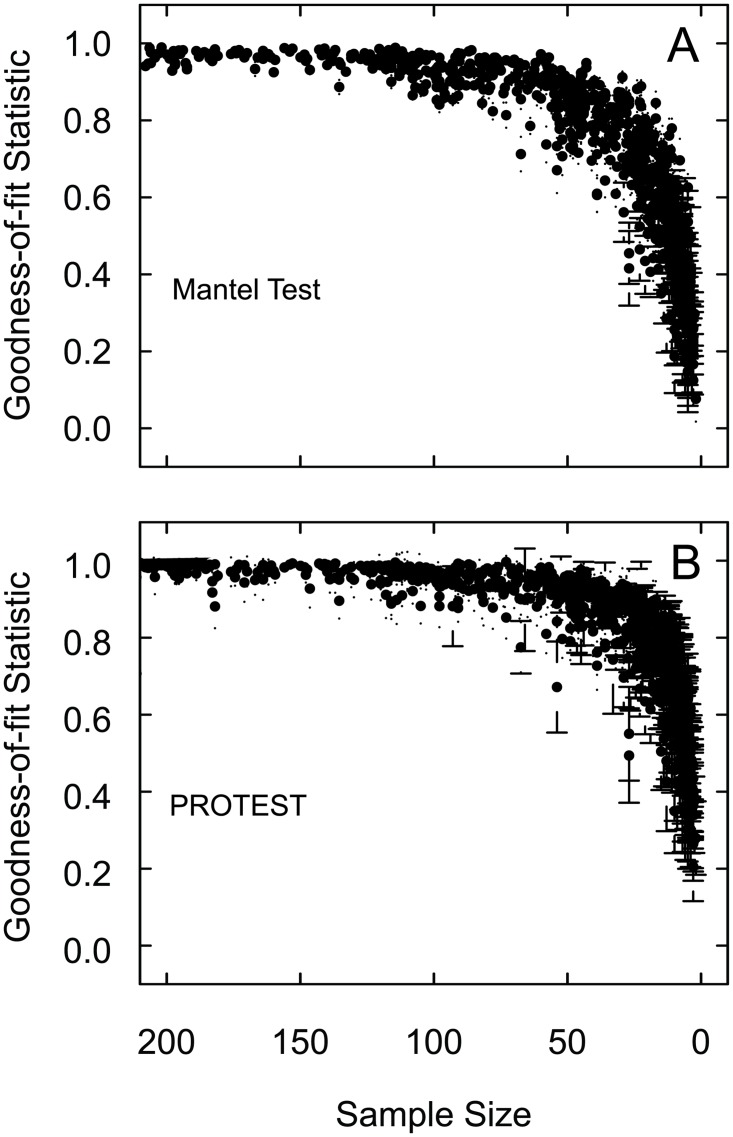
(a) Mantel test and (b) PROTEST comparisons for each of the five median subsample sizes for each of the 220 created datasets with randomly selected abundance structures. Each point (black circles) represents the mean, plus and minus one standard deviation, (a) R-statistic and (b) m^2^-values for the 1000 subsamples of one datasets at one sample size. There are five points for each dataset—one for each of the subsample sizes.

For the 220 datasets with the simulated abundance structure, with the exception of three data points, the Mantel Test R-statistics are greater than R = 0.82 for all sample sizes greater than 48 individuals (Fig 2a). When the median sample size is less than 54 individuals the R-statistics rapidly decrease. The PROTEST m^2^-values are greater than the threshold of R = 0.79 for all sample sizes greater than 50 individuals (Fig 2b). At a median sample size less than 50 individuals the m^2^-values rapidly decrease. No pattern or separation in the goodness-of-fit statistics (both the Mantel Test R-statistics and the PROTEST m^2^-values) was associated with the variables: numbers of taxa, numbers of samples, or initial median sample size (Table 3).

**Table 3:** A list of the results from the various tests of significance used to determine if there were differences in groupings of goodness-of-fit statistics at a sample size 50.

Type of test	Groups being tested	p-value
T-test	High and low evenness dataset R-statistics	p < 0.001
T-test	High and low evenness datasets m2-values	p = 0.003
T-test	R-statistics for the datasets with 5 samples and 10 sample	p = 0.03
T-test	m2-values for the datasets with 5 samples and 10 sample	p < 0.001
ANOVA	R-statistics for the datasets with a richness of 10, 20, and 50	p = 0.006
Bonferroni corrected T-test	R-statistics for the datasets with a richness of 20 and 50	p = 0.009
Bonferroni corrected T-test	R-statistics for the datasets with a richness of 10 and 20	p = 0.053
Bonferroni corrected T-test	R-statistics for the datasets with a richness of 10 and 50	p = 0.94
ANOVA	m2-values for the datasets with a richness of 10, 20, and 50	p = 0.53
T-test	R-statistics the datasets with a richness of 10 and 50 (mixed evenness datasets)	p = 0.09
T-test	m2-values the datasets with a richness of 10 and 50 (mixed evenness datasets)	p = 0.04

These tests were conducted on the 132 created datasets with selected abundance structures because those datasets resulted in the greatest amount of variation among samples sizes. Because of this variation, we used these tests to determine if certain parameters would require larger sample sizes.

**Fig 3:**
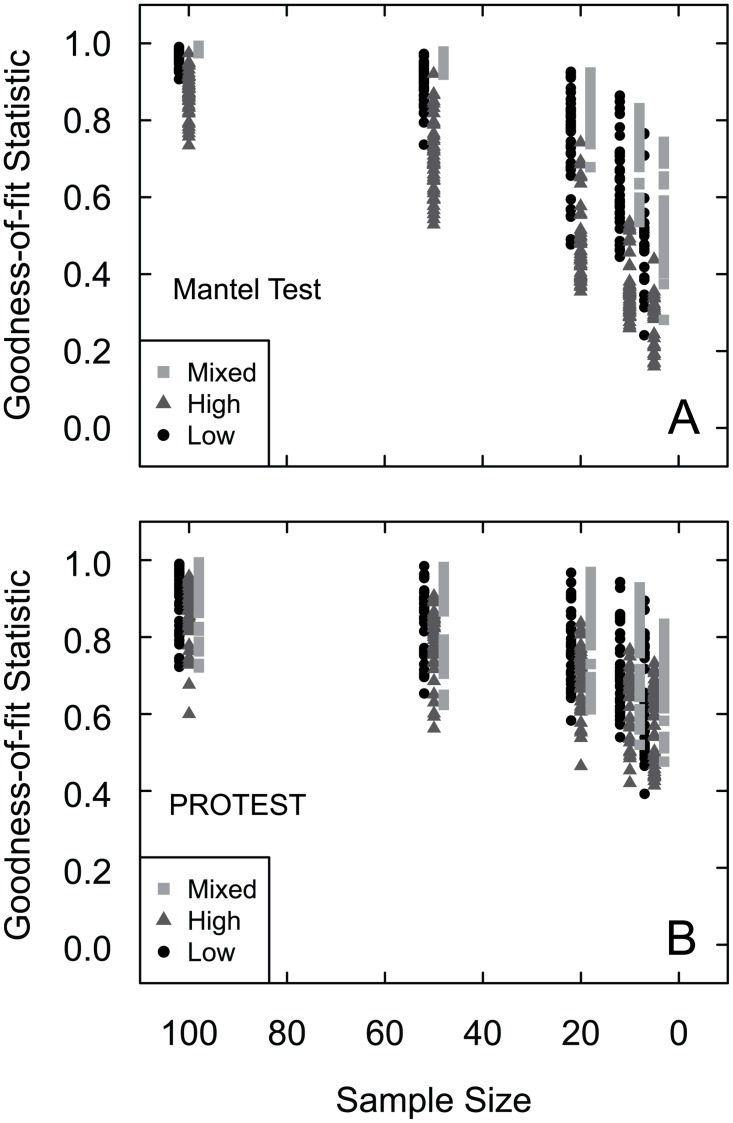
(a) Mantel test and (b) PROTEST comparisons for each of the five median subsample sizes for each of the 132 created datasets with selected abundance structures. Each point (black circles) represents the mean, plus and minus one standard deviation, (a) R-statistic and (b) m^2^-values for the 1000 subsamples of one datasets at one sample size. There are five points for each dataset—one for each of the subsample sizes.

Among the 132 datasets with the selected abundance structure, there was greater variation in the goodness-of-fit statistics compared with the other two dataset types (Fig 3). There was no clear plateau or rapid decrease of goodness-of-fit statistics. Eighty-eight of the datasets were specifically constructed to have either low or high evenness. The 44 low evenness datasets had a mean evenness of 0.58 (Pielou’s J Evenness) [38], and the 44 high evenness datasets had a mean of 0.79. Of these 88 datasets, the low evenness datasets consistently led to greater goodness-of-fit statistic values (Table 4). The mixed evenness datasets, those in which the dataset included some high-evenness samples and some low-evenness samples, produced the highest goodness-of-fit statistics out of these 132 datasets (Table 3; Fig 3). One difference between the PROTEST m^2^-values and the Mantel Test R-statistics was that the mixed evenness datasets’ goodness-of-fit values from the PROTEST were similar to those of the low evenness datasets (Fig 3).

**Table 4:** A list of the minimum and maximum goodness-of-fit statistics for the different parameters for the 132 created datasets with selected abundance structures.

	Mantel Test		PROTEST	
	Min	Max	Min	Max
All 132	0.53	0.98	0.56	0.98
High Evenness	0.53	0.92	0.65	0.98
Low Evenness	0.74	0.97	0.56	0.91
Mixed Evenness	0.92	0.98	0.62	0.98
5 Samples	0.53	0.98	0.70	0.98
10 Samples	0.64	0.97	0.56	0.98
Richness = 10	0.58	0.98	0.65	0.98
Richness = 20	0.53	0.97	0.56	0.97
Richness = 50	0.59	0.97	0.75	0.97
Richness = 10 (mixed evenness)	0.92	0.98	0.65	0.98
Richness = 50 (mixed evenness)	0.92	0.97	0.89	0.98

These goodness-of-fit statistics are for the subsample size of 50.

The ranges and significant differences among the different parameters (evenness and numbers and samples and taxa) vary depending on the parameters and between the Mantel Test and PROTEST (Tables 1 and 2). Of the nine various dataset structures within each set of parameters (for further description, see S1 Supplemental Methods), there is no consistent dataset structure that led to higher goodness-of-fit statistics.

## Discussion

Previously published research collected their sample size based on the probability of ensuring a representative sample of all possible individuals [23,16,24,17,25]. Here, we took a practical approach to determining if smaller sample sizes produce the same results as the originally collected sample size by subsampling 44 previously published and 352 created datasets to five different percentages of the original sample size.

The 44 previously published datasets and 220 of the created datasets demonstrate that smaller sample sizes produce the same multivariate, abundance-based community results as larger sample sizes, in the sense that the results are similar enough that they would be interpreted the same (Figs 1 and 2). Although there were some outliers, the vast majority of the subsample results were above the thresholds when sample sizes were greater than 54 and 58 for the Mantel Test and PROTEST, respectively. This suggests that all median sample sizes greater than these values produced the same results as larger sample sizes As these results are based on median sample sizes within the dataset, a minimum sample-size of 58 individuals per sample would almost certainly be representative for use with these types of multivariate analyses, and as such, 58 individuals is a conservative recommendation for a minimum sample-size to be collected in the field.

This sample size estimate is substantially smaller than found by previous research that used different methods (i.e., probability estimates) to determine that 300 individuals per community are required for ecological research [23,16,24,17, 18,25]. However, the approach taken in the present study of comparing multivariate results is a more practical approach because most community research applies multivariate techniques. Although a smaller sample size may not capture the exact diversity of a community, the smaller sample size would still maintain the general position and order of samples in ordination space as well as the identification of related groupings or gradients of communities when using other multivariate techniques.

Multivariate statistical methods, specifically ordination, may be statistically powerful enough that the differences and similarities among samples are detected even at much smaller samples sizes [39,40]. At these smaller sample sizes, rare taxa may not be collected, and therefore would not be included in the analyses [41]. However, unless the goal of the study is to examine rarity, the results of the present study demonstrate that those rare taxa are not required for interpretation of many ecological results uncovered using multivariate methods.

The median sample size of 58 individuals determined here is less than the combined median sample sizes of the 44 previously published datasets, which was 146 individuals with a range of 10 to 24,812. These 44 datasets are representative of the range of typical median sample sizes collected by ecologists (Table 1). This aids in demonstrating that community studies can collect fewer individuals per sample and still obtain the same meaningful results. This finding is important for managing resources (e.g., time and money, decisions as to where to sample) within a study and for studies where there may only be a limited number of specimens to collect (e.g., small populations and fewer individuals).

The previously published datasets we examined were from a range of environments, geographic locations, and contained a range of taxonomic groups as well as both terrestrial and marine taxa. In addition, the real and created datasets contain a range of numbers of samples, numbers of taxa, and evenness, resulting in datasets spanning an extremely broad range of possible communities. The results were consistent across this broad range of real or realistic communities; none of the above dataset parameters or variables would require larger sample sizes. However, we did not examine methods of tallying taxa other than abundance counts and only multivariate analytical methods were used. So, if methods other than abundance counts and the multivariate statistics employed herein are used, the present study cannot provide insight into the sample size requirements.

In order to strengthen the present results, we recommend future research using a nested sampling protocol (collecting smaller sample sizes within larger) to provide additional information on required sample sizes. Although our data include field-collected datasets, all of the samples sizes smaller than the original were simulated. We did not collect any data at various sample sizes to compare the multivariate statistical results. Future research utilizing nested field sampling may reveal patterns (e.g., patchiness) that would indicate larger sample sizes are required. Conversely, a study of multiple datasets from various environments, locations, and using a range of taxa could provide additional support for the present result that smaller samples sizes produce statistically robust results.

Although we have demonstrated that smaller samples sizes are appropriate for a large range of multivariate ecological research, in many cases, researchers may not be limited in resources, and thus, there would be no real benefit for collecting smaller samples sizes. When resources are not limited, the possibility of over collecting may not be an issue. One instance in which resource-intensive over-sampling may be costly is when studying an endangered species, particularly at the geographic extremes of those species [42,43].

There are many situations in which the costs of identification are high, particularly when species identification requires substantial handling time by the researcher or the number of taxa in a collection locality is low. In such cases, oversampling of individuals results in significantly increased costs, with little improvement in the ability to answer specific ecological questions. For example, taxonomic datasets that were not originally intended to be used for community research may exist that contain a median sample size of 58. With the evidence presented from the present research, ecologists can safely use those data to ask new questions or conduct meta-analyses. These practical implications of the present study demonstrate the most important part of understanding the smallest required sample size for abundance-based, multivariate ecological research.

### When are larger sample sizes required?

#### Evenness

Of the three parameters (number of samples, number of taxa, and evenness) that were systematically varied among the 132 created datasets with selected abundances, evenness had the greatest effect on whether the subsamples of a dataset produced the same multivariate result as the complete dataset. The low evenness datasets had consistently greater goodness-of-fit statistics than the high evenness datasets. There is a significant difference between the low and high evenness datasets for both the R-statistics and m^2^-values; datasets containing samples with consistently high evenness may require larger sample sizes. Thus, when datasets contain samples with consistently high evenness, larger sample sizes are required for detecting similarity and differences among samples in a dataset.

#### Number of taxa

Datasets with more taxa often had lower goodness-of-fit statistics (Tables 3 and 4). However, this pattern was not consistent throughout all numbers of taxa and the two comparison methods. Overall, this effect of the number of taxa on the required sample size is minor relative to the complete analysis of all 396 datasets. In addition to the effect of the number of taxa on selected-created dataset, 11 of the previously published datasets and 50 of the simulated-created datasets had more than 50 taxa, and all of these datasets produced consistently high goodness-of-fit statistics between subsampled and corresponding complete datasets (Tables 1 and 2). So, the majority of datasets, even those with a larger number of taxa, still demonstrate that smaller samples sizes are sufficient for multivariate community research.

#### Number of samples

There was a significant difference in results between 132 selected abundances datasets with 5 and those with 10 samples (Tables 3 and 4; Fig 3). The datasets were constructed so that each sample within the each of these 132 datasets represents a different community. With a greater number of communities (10 versus 5), the multivariate analysis is more likely to distinguish between most of the communities even with fewer individuals per sample. The multivariate analyses are better able to distinguish between the two dichotomous groups of samples, even at smaller sample sizes, which was likely the cause for the high goodness-of-fit statistics. When the datasets are limited to 5 communities, there is less of a chance that the community gradient will still be apparent when sample sizes decrease. When there are 10 communities, there is a higher probability that the relative order of one or two communities will remain intact even at smaller sample sizes, producing the same or similar community gradient in ordination space.

This is additional evidence that homogeneity of communities within a dataset may require larger sample sizes. However, it should be noted that many, if not most, studies seek to determine the cause of community change, so they deliberately sample along suspected gradients or between environmental conditions known to be different. Environmental homogeneity among sampled communities is not a common goal. So, this issue of larger sample size requirements among homogeneous communities should not have a grave impact on community research.

## Conclusion

The primary goal of this study was to determine if smaller sample sizes produce the same results as larger, more typically collected sample sizes. Examining 44 previously published and 220 created datasets with simulated abundance structures, we found evidence that smaller sample sizes (i.e., 58 individuals) produce the same community results as larger sample sizes.

This finding is most important for ecologists with limited resources (e.g., money, time, or the data). Many ecology researchers are underfunded. Money spent on field collections and research assistantships can, if limited, still obtaining meaningful statistical information because smaller samples sizes are sufficient to accomplish the same research goals.

To detect possible dataset parameters that require larger sample sizes, we subsampled selected-created datasets in which the number of samples, number of taxa, and evenness were systematically altered to test for an effect on the required sample size. We found that high evenness datasets produced lower goodness-of-fit statistics than low evenness and mixed evenness datasets. Although high evenness datasets may have led to lower goodness-of-fit, few studies would consist entirely of uniformly high-evenness communities.

## Supporting Information

S1 Fig Visual representation of the synthetic dataset simulation process using 5 taxa and 5 samples. Each of the five normal distributions represents the simulated, possible distribution of on taxon along that environmental gradient. Each of the five black dashed lines represents five randomly selected locations along the environmental gradient where samples were selected, representing the fossil collection. For each normal distribution that crossed each sample line a random select of abundance was selected, represented by the dashed gray line.
(EPS)

S1 R Data List of the created datasets with selected abundances. A R-formatted file containing the 132 created datasets with selected abundance distributions.
(TXT)

S1 Supplemental Methods Supplemental methods section. A more detailed description of the methods used to develop the 132 created datasets with selected abundance distributions.
(DOCX)
