# Supplementary material for: Reexamining Sample Size Requirements for Multivariate, Abundance-Based Community Research: When Resources are Limited, the Research Does Not Have to Be
Source: PLoS One. 2015 Jun 9;10(6):e0128379. doi: 10.1371/journal.pone.0128379 (PMC4461312; doi:10.1371/journal.pone.0128379)
Supplement: S1 Supplemental Methods — A more detailed description of the methods used to develop the 132 created datasets with selected abundance distributions. (DOCX) [file pone.0128379.s003.docx]

*Supplemental Description of the Selected-Created Dataset Construction*

We systematically varied the number of samples, richness, and evenness when creating the 132 selected-created datasets in order to examine these three parameters as possible influences on the required sample size for community research. By varying the number of samples in each dataset, we defined 81 datasets containing 5 samples and 51 datasets containing 10 samples. Five and 10 were chosen in order to keep the datasets simple while still being able to evaluate whether the number of samples had an effect on the required sample size. The 5- or 10-sample datasets represent real datasets of 5 or 10 different communities, respectively. In some community studies, some samples will have a similar enough taxonomic makeup to be indistinguishable in a multivariate analyses; these samples would be considered the same community. Because all 5 or 10 samples in the 132 constructed datasets have a different taxonomic makeup and are designed to be distinguishable in multivariate analyses, each of the 5 or 10 samples represents a distinct community.

The second parameter that was varied among the 132 datasets was taxonomic richness within each sample. Within the datasets of 5 samples, 27 have a richness of 10, 27 have a richness of 20, and 27 have a richness of 50. Within the datasets of 10 samples, 24 have a richness of 10 and 27 have a richness of 20.

The third parameter varied among the 132 datasets was the evenness. There were three evenness categories: (1) the 44 low evenness datasets, with individual-sample evenness values ranging from 0.14 to 0.98 and a mean evenness of 0.58 (Pielou’s J Evenness; Magurran 1998); (2) the 44 high evenness datasets with individual-sample evenness values from 0.18 to 1.0, with a mean of 0.79; and (3) the 44 mixed evenness datasets, with each dataset containing individual samples of both high and low evenness, with individual-sample evenness values from 0.13 to 1.0, with a mean of 0.69. Each sample in each dataset had its evenness varied by starting with one taxon with the greatest abundance while the remaining taxa all had an abundance of 1. For example, a dataset with 5 samples and 10 taxa would have one sample with one taxon having a sample of 191 while the remaining taxa all had an abundance of 1 (Supplemental datasets). This example would have been an extreme case of low evenness. Each abundance distribution in each sample of each dataset systematically varied until a dataset contained one sample with every taxon having the same abundance. This example would have been an extreme case of high evenness.

A set of parameters incorporates the three controlled parameters (samples, richness, and evenness). An example set of parameters is 5 samples, a richness of 10, and low evenness. The two sample parameters (5 and 10), the three richness parameters (10, 20, and 50), and the three evenness parameters (high, low, and mixed) combined to create 16 unique sets of parameters. These 16 sets lead to 16 primary datasets. Each primary dataset was set up as a typical community ecology dataset, with a series of samples each containing a number of taxa with varying abundances. The 16 primary datasets do not contain any taxonomic abundances of zero in any of the samples. No datasets with zero were included in order to keep the dataset variants relatively simple; once an abundance of zero was added for any taxon in any sample, it would have had to be systematically varied throughout. This would have led to an exponentially greater number of datasets requiring subsampling, and therefore, an exponentially greater amount of time added to the study.

Various dataset structures were created in order to examine the effect that changing a particular dataset with respect to a certain set of parameters has on the required sample size. A dataset structure*,* for the purposes of this study, refers to the variation of the 16 sets of parameters created by varying taxonomic placement within a sample. Each of these structures creates a variant of the 16 primary datasets, to produce 8 to 9 different variations of the 16 primary datasets equaling 132 total datasets (all 132 datasets can be found within the Supplemental Material). The first dataset structure was simply the primary dataset of each of the 16 sets of parameters. Each of the remaining 7 or 8 dataset structures manipulated the primary dataset structure in some manner.

The second structure was created by varying the presence or absence of the single most abundant taxon within each sample of the first structure (primary dataset). For example, within the primary dataset that consists of 5 samples, a richness of 10, and low evenness, the same taxon is the most abundant taxon in all five samples. To create the second structure, an additional four taxa are added to the dataset of the first structure. For each of the four new taxa, an abundance of zero is added to four samples, while in the fifth sample, the value of the highest taxonomic abundance of that sample is added. The result is four new samples, each with one new taxon replacing the original most abundant taxon, and one unchanged sample (with an abundance of zero for all four new taxa).

For the next two structures (the third and fourth), the remaining taxa in each sample—all but the most abundant taxon—were divided into thirds. The first third contained the moderately abundant taxa, and the second two-thirds contained the rare taxa.

For the third structure, the moderately abundant taxa were varied in the same manner as the most abundant taxon in the second structure. The difference between the second and third structures is all of the taxa varied in concert, as opposed to one taxon at a time; each group of three taxonomic abundances was either present or absent in the remaining samples. The most abundant taxon and the rare taxa remained constant through all the samples in the datasets with this structure.

For the fourth structure, the rare taxa were varied in the same manner as the most abundant taxon in the second structure and the moderately abundant taxa of the third structure. The most abundant taxon and moderately abundant taxa remained constant through all the samples in the datasets with this structure.

The fifth and sixth structures changed the position of the most abundant taxon through the five or 10 samples. The most abundant taxon would change from taxon A to taxon B from sample 1 to sample 2 and from taxon A to taxon C from sample 1 to sample 3. The reason this manipulation was occasionally done to two different structures (fifth and sixth or just fifth) was because datasets with only 5 samples and 10 taxa could only have the most abundant taxon vary through 5 different locations. The sixth structure placed the most abundant taxa in the position once occupied by taxa with lower abundances. The sixth structure is the one structure that was not possible to create for all the different parameters. When there were 10 samples and 10 taxa, switching the most abundant taxa would take up all of the taxa with just one structure.

The final three structures were all randomized dataset constructions. The position of each taxon’s abundance from the primary dataset (the first structure) was randomly placed in any one of the taxonomic positions. The difference between the three structures was the number of zeros available to add and the total number of taxa. Structure seven contained no zeros with the same number of taxa as the first six structures. Structure eight added an additional possible 5, 10, or 15 zeros for the 10, 20, and 50 taxa datasets, respectively, with the same number of taxa as the first six structures. The ninth and final structure added an additional possible 5, 10, or 15 zeros for the 10, 20, and 50 taxa datasets, respectively, and also added that number of taxa to the dataset so no abundance was unused.
